# Efficiency of transurethral en‐bloc resection vs. conventional transurethral resection for non‐muscle‐invasive bladder cancer: An umbrella review

**DOI:** 10.1002/cam4.7323

**Published:** 2024-05-31

**Authors:** Deng‐xiong Li, Qing‐xin Yu, Rui‐cheng Wu, Jie Wang, De‐chao Feng, Shi Deng

**Affiliations:** ^1^ Department of Urology Institute of Urology, West China Hospital, Sichuan University Chengdu Sichuan Province China; ^2^ Department of pathology Ningbo Clinical Pathology Diagnosis Center Ningbo City Zhejiang Province China; ^3^ Division of Surgery & Interventional Science University College London London UK

**Keywords:** bladder cancer, En bloc resection of bladder tumor, transurethral resection of bladder tumor, urothelial carcinoma

## Abstract

**Background:**

En‐Bloc transurethral resection of bladder tumor (ERBT) was clinically used to resect non‐muscle‐invasive bladder cancer (NMIBC). However, discrepancies persist regarding the comparisons between ERBT and conventional transurethral resection of bladder tumor (cTURBT).

**Methods:**

We conducted a comprehensive search in PubMed, Embase, Web of Science, Cochrane Database of Systematic Reviews, and performed manual searches of reference lists to collect and extract data. Data evaluation was carried out using Review Manager 5.4.0, Rx64 4.1.3, and relevant packages.

**Results:**

There were nine eligible meta‐analyses and nine eligible RCTs in our study. NMIBC patients undergoing ERBT were significant associated with a lower rate of bladder perforation and obturator nerve reflex compared to those receiving cTURBT. Our pooled result indicated that ERBT and cTURBT required similar operation time. Regarding postoperative outcomes, ERBT demonstrated superior performance compared to cTURBT in terms of detrusor muscle presence, catheterization time, and residual tumor. ERBT exhibited a higher rate of three‐month recurrence‐free survival (RFS) compared to those receiving cTURBT (*p* < 0.05; I^2^ = 0%). In bipolar subgroup, ERBT had a significant better 12‐month RFS than cTURBT (*p* < 0.05; I^2^ = 0%). Simultaneously, the exclusion of Hybrid Knife data revealed a significant improvement in 12‐month RFS associated with ERBT (*p* < 0.05; I^2^ = 50%).

**Conclusion:**

Using a combination of umbrella review and meta‐analysis, we demonstrated that ERBT had better or comparable perioperative outcome and improved 3 and 12 month RFS than cTURBT. We suggest that ERBT maybe a better surgical method for patients with NMIBC compared with cTURBT.

## 1 INTRODUCTION

Ranked as the 6th most prevalent cancer, bladder cancer (BCa) stands as the 9th leading cause of cancer‐related deaths in men.^1^ Around 70% of newly diagnosed BCa patients present with non‐muscle‐invasive bladder cancer (NMIBC), known for its high recurrence and progression rates.^2^, ^3^ Various approaches have been employed to enhance the perioperative and survival outcomes of NMIBC patients.^4^, ^5^ Approximately 30% of patients diagnosed with NMIBC experience recurrence within 12 months after conventional transurethral resection of bladder tumor (cTURBT) and intravesical instillation.^6^, ^7^ The need for repeated cTURBT places a significant economic and physical burden on these patients. Additionally, a notable percentage of these patients may advance to muscle‐invasive BCa, which is linked to poor overall survival rates despite undergoing radical cystectomy and adjuvant therapy.^8^, ^9^ Thus, many treatments are exploring to improve the prognosis of patients with NMIBC.^4^, ^10^, ^11^

En‐Bloc transurethral resection of bladder tumor (ERBT) was initially reported in 1997.^29^ However, this technique did not attract the attention of most of researchers until 2011. Herrmann et al.^12^ improved the technique of ERBT and reported the positive outcomes of six NMIBC patients. Meanwhile, ERBT can offer a complete specimen, thereby facilitating the investigation of the tumor microenvironment.^13^, ^14^ These outstanding findings have prompted further studies on ERBT in many teams. Multiple studies have identified ERBT as a developing alternative to cTURBT. In fact, the European Association of Urology recommends the utilization of ERBT for resecting NMIBC.^15^ However, discrepancies persist regarding the comparisons between ERBT and cTURBT. In 2020, Teoh et al.^16^ reported no statistically significant difference in recurrence‐free survival (RFS) between ERBT and cTURBT based on pooled data up until June 2019. In 2023, Teoh et al.^17^ demonstrated NMIBC patients undergoing ERBT were significantly associated with longer RFS than those receiving cTURBT based on the results of a multicenter randomized trial (EB‐STAR study). Furthermore, surrounding perioperative outcomes, there are still some controversies between ERBT and cTURBT. For instance, most meta‐analyses^18^, ^19^, ^20^, ^21^, ^22^ indicated that ERBT and cTURBT required similar operation times. However, one meta‐analysis reported that ERBT would require more time for tumor resection.^16^

In this study, our objective was to tackle these issues by conducting an umbrella review of ERBT in NMIBC. Additionally, we carried out a pooled analysis using data from randomized controlled trials (RCTs) to reconcile discrepancies noted in various meta‐analyses.

## 2 MATERIALS AND METHODS

We conducted an umbrella review of early recurrence bladder tumors in NMIBC following the Preferred Reporting Items for Systematic Reviews and Meta‐analyses (PRISMA) guidelines.^23^

### 2.1 Literature search

In May 2023 (last update), we systematically searched PubMed, Embase, Web of Science, and the Cochrane Database of Systematic Reviews to identify relevant systematic reviews, meta‐analyses, and RCTs. Referring to the Scottish Intercollegiate Guidelines Network”s guidance,^24^ a comprehensive literature search on ERBT was conducted using a combination of Medical Subject Headings terms, keywords, and various text word variations across multiple databases. The search terms included (en‐bloc resection OR ERBT OR ETURBT) AND (bladder tumor). Initially, two authors (DXL and DCF) independently screened titles and abstracts retrieved from the databases. Subsequently, meta‐analyses and RCTs meeting the inclusion criteria were identified through full‐text reading by the two authors. In cases of discrepancies, a third author (RCW) resolved the differences in literature screening. Additionally, a manual search was performed to review the meta‐analyses, reviews, and RCTs cited in the references of selected articles.

### 2.2 Study selection

We examined the efficiency of ERBT and cTURBT in terms of perioperative outcomes and survival benefits. The systematic reviews and meta‐analyses included in the analysis had to meet specific criteria: they had to be systematic reviews of RCTs or cohort studies, case–control studies, or cross‐sectional studies comparing the efficiency of ERBT and cTURBT. The RCTs included in the analysis had to meet certain criteria as well: they had to compare ERBT and cTURBT, have accurate and available data on perioperative outcomes and survival benefits. Studies in languages other than English, as well as animal and cell culture studies, were excluded.

### 2.3 Data extraction

The following information was independently extracted from each included meta‐analysis by two reviewers (DXL and DCF): (1) first author”s name, (2) publication year, number of included studies and patients, (3) perioperative outcomes and (4) RFS. Any disagreement was determined by a third author (RCW).

The information extracted from each RCT included the first author”s name and publication year, country of the study, energy for ERBT and cTURBT, patient numbers, tumor size, tumor number, T stage, World Health Organization (WHO) grade, inclusion of carcinoma in situ (CIS), adjuvant medicine type, estimated summary effect (risk ratio (RR), odds ratio (OR), mean difference (MD), standardized summary effect (SMD) with 95% confidence intervals (CI)), heterogeneity (I^2^), perioperative outcomes (operation time (ORT), bladder irrigation), and RFS at 3 and 12 months. In cases where a RCT was published as both an article and conference paper, data from the most recent study was prioritized. Any disagreements were resolved by a third author.

### 2.4 Quality assessment of methods and evidence

ROBIS^25^ was used to evaluate methodological quality of the included meta‐analyses by two reviewers (DXL and DCF). ROBIS consisted of three phases, with results being rated as low, high, or unclear. Additionally, each health outcome underwent evidence evaluation and was assigned a quality grade of “high,” “moderate,” “low,” or “very low” based on the Grading of Recommendations, Assessment, Development, and Evaluation (GRADE).^26^

Two reviewers (DXL and DCF) independently assessed the methodological quality of the RCTs included in our meta‐analysis following the guidelines outlined in the Cochrane Handbook.^27^ According to the result, we classified the studies into one of the three levels: low risk of bias, unclear risk of bias, or high risk of bias. Any disagreement was determined by a third author (RCW).

### 2.5 Statistical analysis

The data was evaluated using Review Manager 5.4.0, Rx64 4.1.3, and their respective packages. Continuous outcomes were assessed using MD with 95% CI, while dichotomous outcomes were assessed using OR with 95% CI. A random‐effects model was applied for data analysis in the presence of significant heterogeneity (*p* < 0.05), with heterogeneity evaluated using the I^2^ statistic where I^2^ >50% indicated high heterogeneity. The statistical significance level was set at *p* <0.05.

## 3 RESULTS

**FIGURE 1:**
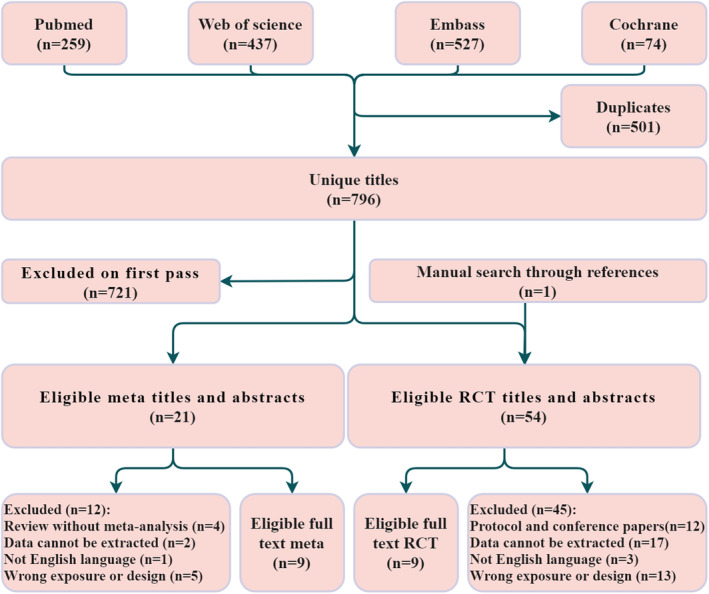
Work flow diagram.

A total of 1295 studies were initially identified through database searches. After removing duplicates, 797 studies were screened, resulting in the selection of 21 studies for potential inclusion in meta‐analyses and 54 studies for potential inclusion in RCTs. Ultimately, nine meta‐analyses and nine RCTs met the eligibility criteria for our study (Figure 1). The studies included in each meta‐analysis are presented in Table S1.

### 3.1 The characteristics of eligible studies and risk of bias assessment

Table 1 showed the characteristics of eligible meta‐analyses.^16^, ^18^, ^19^, ^20^, ^21^, ^22^, ^28^, ^29^, ^30^ Out of the eligible meta‐analyses, two studies exclusively incorporated RCTs.^16^, ^20^ The latest search day was January 2022.^20^ Table 2 contained the characteristics of eligible RCTs.^17^, ^31^, ^32^, ^33^, ^34^, ^35^, ^36^, ^37^, ^38^ Four of these RCTs came from China, two from Egypt, one from Romania, one from Germany and one from Spain. Five RCTs selected holmium laser as energy for ERBT group, two selected bipolar, one RCT selected green‐light laser and one selected Hybrid Knife.

**TABLE 1:** Summary of included meta‐analyses and outcomes.

Author (Year)	Last research	Included studies	Type	No. of EBRT	No. of cTURBT	Outcomes
Wang_CW (2023)	April 2021	31	RCT/NRCT	2024	2171	RTR, detrusor muscle, RFS (same‐site, 3, 12, 24 months), ORT, HPT, CTT, bladder perforation, ONR, bladder irritation
Yanagisawa_T (2022)	August 2021	29	RCT/NRCT	4484		RTR, RFS (12, 24 months), ORT, CTT, bladder perforation, detrusor muscle, muscularis mucosa, CIS
Motlagh_RS (2022)	June 2021	14	RCT/NRCT	2092		RFS (3 and 12 months), detrusor muscle, SAER
Li_ZY (2022)	January 2022	7	RCT	1870	1844	RTR, ORT, HPT, CTT, Re‐TURBT, ONR, bladder perforation, Hemoglobin deficit, Detrusor muscle, Urethral stricture, RFS (3, 12, 24 and 36 months)
Di_Y (2022)	January 2022	28	RCT/NRCT	1142		RFS (12 and 24 months), HPT, CTT, bladder irritation
Zhang_D (2020)	November 2019	19	RCT/NRCT	1870	1844	RFS (12 and 24 months)
Yang_H (2020)	April 2019	9	RCT/NRCT	1020		ORT, HPT, CTT, bladder irrigation, RFS (24 months), bladder perforation, ONR, Urethral stricture, postoperative adjuvant intravesical chemotherapy
Wu_YP (2016)	September 2016	7	RCT/NRCT	438	448	ORT, HPT, CTT, RFS (24 months), ONR, bladder perforation, bladder irritation, urethral stricture, postoperative adjuvant intravesical chemotherapy
Teoh_YJ (2020)	June 2019	13	RCT	586	569	ORT, bladder irritation, CTT, HPT, ONR, bladder perforation, detrusor muscle, RFS (12, 24 and 36 months)

Abbreviations: CTT, catheterization time; CTURBT, conventional transurethral resection of bladder tumor (CTURBT); EBTR, En‐Bloc transurethral resection of bladder tumor; HPT, hospitalization time; NRCT, non‐randomized controlled trial; ONR: obturator nerve reflex; ORT: operation time; PFS, Progression‐free survival; RTR, residual tumor rate; RFS, recurrence‐free survival; SAER: serious adverse event rates; RCT, randomized controlled trial.

**TABLE 2:** The characteristics of included RCTs.

Author	Year	Country	Energy	No. sample (EBRT/cTURBT)	Tumor size	No. tumor	T stage	WHO grade	CIS	Adjuvant therapy
BĂLAN_GX	2018	Romania	Bipolar vs. monopolar	45/45	<=3 cm	NA	Ta/T1	G1_3	NA	Epirubicin/BCG
Liu_H	2013	China	2‐mm (thulium) laser vs. monopolar	64/56	NA	NA	Ta/T1	PUNLM, low, and high	NA	Epirubicin
Chen_X	2014	China	2‐mm (thulium) laser vs. monopolar	71/71	NA	NA	Ta/T1	PUNLM, low, and high	Yes	Epirubicin
Gakis_G	2020	Germany	Hybrid Knife vs. monopolar	56/59	>0.5 cm	<=5	Ta/T1	G1_3	Yes	Mitomycin C/BCG
Hashem_A	2021	Egypt	Holmium Laser vs. monopolar	42/49	<=5 cm	<=5	Ta/T1	G1_3	Yes	Epirubicin
Fan_JH	2021	China	Green‐light laser vs. monopolar	116/117	<=3 cm	NA	Ta/T1	PUNLM, low, and high	Yes	Pirarubicin
Teoh_JY	2023	China	Bipolar vs. bipolar	143/133	<=3 cm	NA	Ta/T1	PUNLM, low, and high	Yes	Mitomycin C
Gallioli_A	2022	Spain	Holmium Laser/monopolar/bipolar vs. monopolar/bipolar	140/108	<=3 cm	<=3	Ta/T1	PUNLM, low, and high	Yes	Mitomycin C/Epirubicin
Badawy_A	2022	Egypt	2‐mm (thulium) laser vs. monopolar	60/60	NA	<=2	Ta/T1	Low and high	NA	Doxorubicin

Abbreviations: No, number of; CIS, carcinoma in situ; BCG: Bacillus Calmette‐Guerin; NA, no data; WHO, World Health Organization.

**FIGURE 2:**
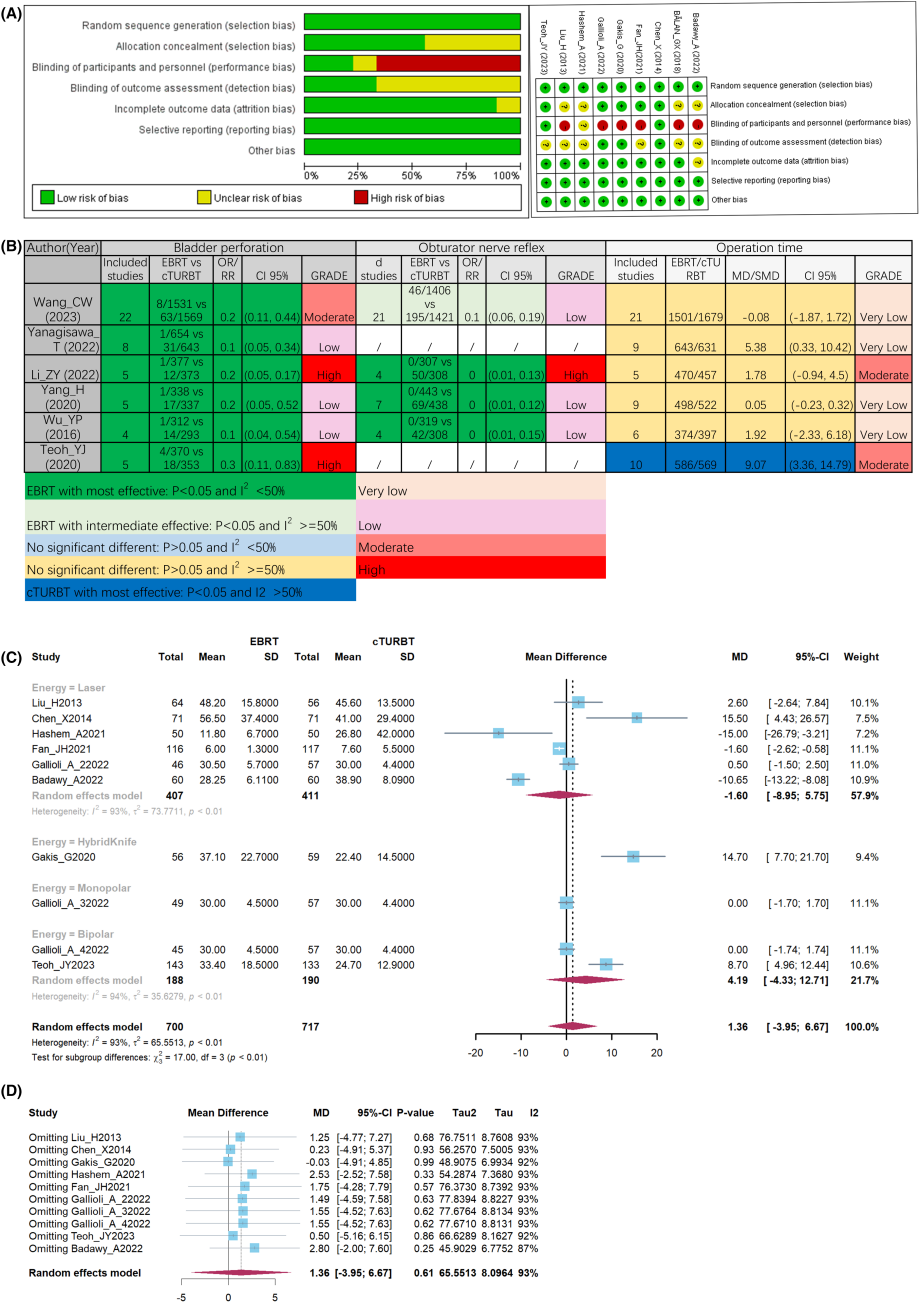
Quality assessment of included RCTs (A), outcomes during operation from meta‐analyses (B), pooled result (C), and sensitivity analysis, (D) of operation time.

According to the results of risk of bias assessment, only two of nine (22.2%) meta‐analyses were low risk (Table S2). Four of nine (44.4%) were classified as high risk due to English limit in searching section. For RCTs, six of nine (66.7%) had performance bias due to the surgery was hardly to performed blind method (Figure 2A).

### 3.2 ERBT has comparable results during operation

All identified meta‐analyses consistently reported a significantly lower incidence of bladder perforation in patients who underwent ERBT compared to those who underwent cTURBT (Figure 2B). Table S3 contained the detail of GRADE assessment. Similarly, patients received ERBT was statistically associated with lower rate of obturator nerve reflex (ONR) than those accepted cTURBT (Figure 2B). In term of ORT, there was no difference between ERBT and cTURBT in five meta‐analyses.^18^, ^19^, ^20^, ^21^, ^22^ Only Teoh et al^16^ found that ERBT had longer ORT. Consequently, we synthesized the data from eight RCTs pertaining to ORT. Among these RCTs, Gallioli et al.^36^ conducted four separate comparisons based on variations in energy levels. From Figure 2C, no significant difference was observed between ERBT and cTURBT in terms of ORT (MD: 1.36; 95% CI: −3.95, 6.67; p > 0.05). However, substantial heterogeneity was observed (I^2^ = 93%), which was consistence with previous meta‐analyses.^18^, ^19^, ^20^, ^21^, ^22^ We were failed to find out the cause of heterogeneity, despite conducting subgroup analyses based on energy source and country. In further sensitivity analysis, no significant decrease of heterogeneity was observed (Figure 2D).

### 3.3 ERBT has better postoperative results than cTURBT

**FIGURE 3:**
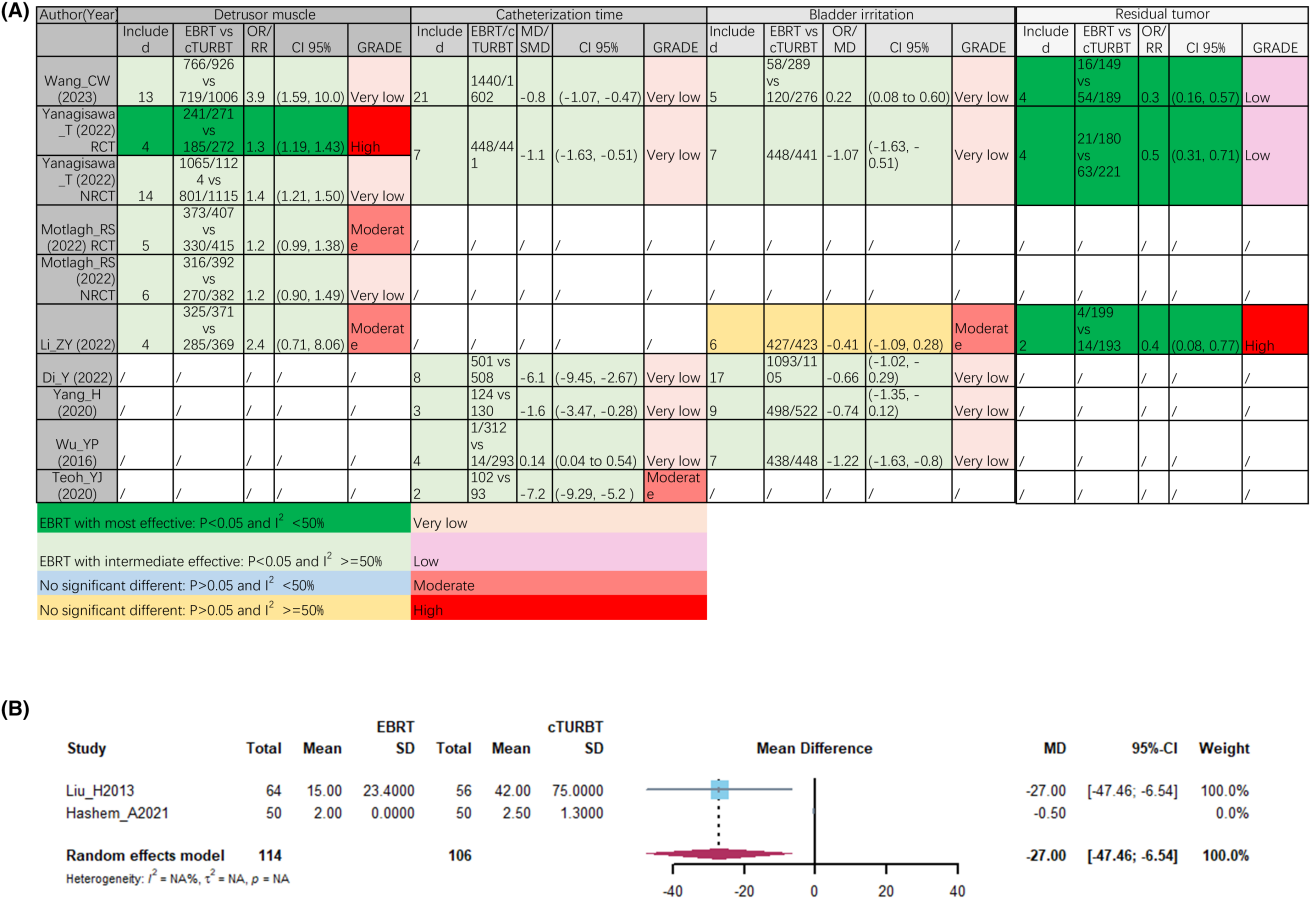
Postoperative outcomes from meta‐analyses, (A) pooled result of bladder irritation (B).

In postoperative outcomes, four meta‐analyses with six separate comparisons consistently identified that ERBT yielded a higher rate of detrusor muscle acquisition compared to cTURBT (Figure 3A). Similarly, NMIBC patients received ERBT had shorter catheterization time (CTT) and a lower rate of residual tumor than those accepted cTURBT. Regarding bladder irrigation, five meta‐analyses consistently reported that ERBT was associated with either a shorter duration or a lower rate of bladder irritation compared to cTURBT. However, Li et al.^20^ did not find significant difference in bladder irritation between these two groups. Thus, we tried to synthesized the data from RCTs pertaining to bladder irritation. Unfortunately, only two RCTs provided data and one of them could not be calculated (Figure 3B). At least, ERBT and cTURBT exhibited comparable outcomes of bladder irritation.

### 3.4 ERBT may bring survival benefits to NMIBC patients

**FIGURE 4:**
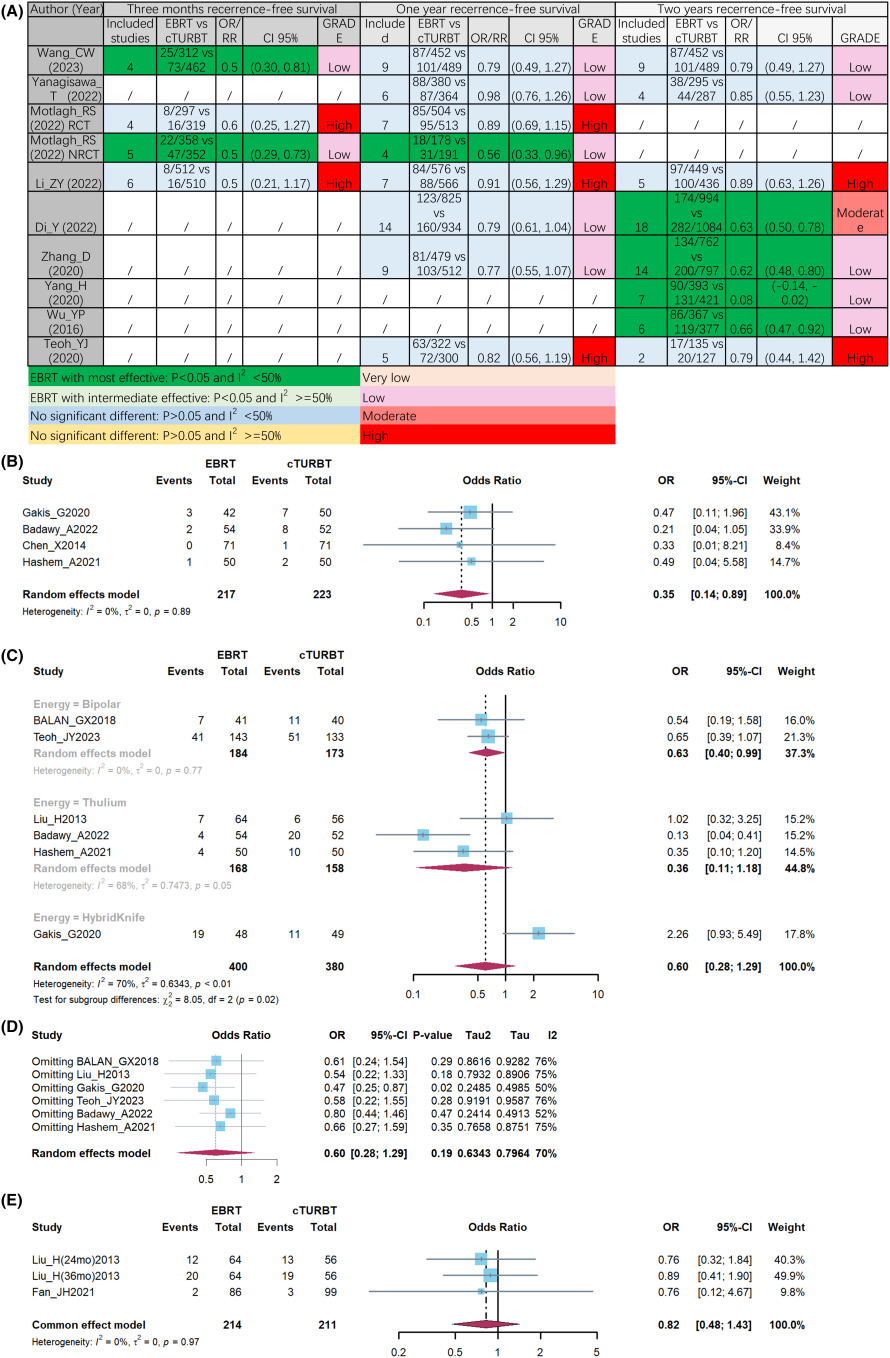
Recurrence‐free survival (RFS) outcomes from meta‐analyses (A), pooled result of 3‐month RFS (B), pooled result (C), and sensitivity analysis (D), of 12‐month RFS, pooled result of more than 12‐month RFS (E).

In a 3‐month period, two comparisons showed positive outcomes for patients with NMIBC treated with ERBT, while two other comparisons found no significant difference between ERBT and cTURBT (Figure 4). When looking at 12‐month RFS, only one out of eight comparisons showed a survival benefit with ERBT, while the remaining seven comparisons found no significant difference in RFS between ERBT and cTURBT. For 24‐month RFS, half of the meta‐analyses indicated a significant survival benefit with ERBT, while the other four did not find statistical survival benefits. These combined results highlight a debate over whether ERBT can offer significant survival advantages to NMIBC patients.

Consequently, pooling the results of four RCTs, we observed that patients undergoing ERBT exhibited a higher rate of 3‐month RFS compared to those receiving cTURBT (Figure 4B; OR: 0.35; 95% CI: 0.17, 0.89; *p* < 0.05; I^2^ = 0%). In terms of 12 months RFS, there was no significant difference between ERBT and cTURBT based on all meta‐analyses (Figure 4C; OR: 0.6; 95% CI: 0.28, 1.29; *p* > 0.05; I^2^ = 70%). To find the cause of heterogeneity, we conducted subgroup analyses based on energy source. Then, in bipolar subgroup, ERBT had a significant better 12‐month RFS than cTURBT (Figure 4C; OR: 0.63; 95% CI: 0.4, 0.99; *p* < 0.05; I^2^ = 0%). During the sensitivity analysis, the exclusion of Gakis_G et al.”s^35^ data resulted in a decrease in I^2^ to 50%. Simultaneously, the omission of data from the Hybrid Knife revealed a significant improvement in 12‐month RFS associated with ERBT (Figure 4D; OR: 0.47; 95% CI: 0.25, 0.87; *p* < 0.05; I^2^ = 50%). Therefore, based on our findings, we propose that NMIBC patients who undergo ERBT, particularly with the exclusion of Hybrid Knife energy, exhibit improved 3 and 12 month RFS, especially when utilizing bipolar energy. In RFS longer than 12 months, there was no significantly difference between ERBT and cTURBT (Figure 4E, OR: 0.82; 95% CI: 0.48, 1.43; *p* = 0.97; I^2^ = 0%).

## 4 DISCUSSION

In 1997, Kawada et al.^39^ initially reported the clinical application of ERBT with monopolar arched electrode. However, some controversies remain unresolved and require further investigation. Recently, these problems are discussed in several studies (including meta‐analysis and RCT).^17^, ^22^, ^31^ Using a combination of umbrella review and meta‐analysis, we demonstrated that ERBT had a better or comparable perioperative outcome than cTURBT. Furthermore, NMIBC patients undergoing ERBT exhibited improved 3 and 12 month RFS compared to those receiving cTURBT. We suggest that ERBT maybe a better surgical method for patients with NMIBC compared with cTURBT.

When compared to cTURBT, ERBT has shown a notable association with reduced rates of bladder perforation and ONR, suggesting that it may offer a safer surgical alternative for NMIBC patients.^16^, ^18^, ^19^, ^20^, ^21^, ^22^ The pooled ORT result reported by Teoh et al.^16^ exhibited disparities from the findings of other five meta‐analyses.^18^, ^19^, ^20^, ^21^, ^22^ In our meta‐analysis, comprised of five studies, ERBT and cTURBT required a similar amount of time to complete the surgery. Surgeons may need to invest time in learning a new surgical technique, which could explain the significant heterogeneity observed across different meta‐analyses. Considering these results, we tentatively suggest that ERBT is a safe surgical option.

Invasive detrusor muscle is the diagnostic criterion for NMIBC and MIBC.^15^ Simultaneously, obtaining the entire tumor specimen could assist pathologists in making more accurate diagnoses and facilitate the identification of histological variants that had a significant impact on the prognosis of patients with NMIBC.^40^, ^41^, ^42^ Moreover, all pooled meta‐analyses demonstrated that ERBT was associated with a lower incidence of residual tumor. These two pieces of evidence confirmed the sufficient efficacy of ERBT in managing NMIBC. In term of postoperative outcomes, patients undergoing ERBT had statistically shorter CTT based on six meta‐analyses.^16^, ^18^, ^19^, ^21^, ^29^, ^43^ Li et al.^20^ did not find significant difference in bladder irritation between these two groups, while other five meta‐analyses identified a significant association between ERBT and shorter duration of bladder irritation.^16^, ^18^, ^19^, ^21^, ^22^ Consistent to the five meta‐analyses, Liu et al.^38^ also reported a significant correlation between ERBT and shorter bladder irritation. Hashem et al.^37^ found two patients in cTURBT group had bladder irritation, while no patients in ERBT group diagnosed bladder irritation. These results revealed that ERBT offered a comparable or even better performance of bladder irritation.

Almost 30% of NMIBC patients would experience recurrence even after accepting BCG.^15^, ^44^ Therefore, researchers strived efforts to find powerful biomarkers and various new therapies to improve the prognosis of patients with NMIBC.^45^, ^46^ Urological surgeons have widely deliberated on the potential of ERBT to enhance RFS in patients with NMIBC.^47^ However, no consensus has been reached on this matter now. Controversies arose regarding the 3, 12, and 24‐month RFS based on the pooled results of the aforementioned meta‐analyses. Thus, we collected and pooled the data on 3‐month RFS, which revealed that patients in the ERBT group exhibited superior RFS compared to those in the cTURBT group. In the result of 12‐month RFS, ERBT with bipolar had significant better RFS. After excluding the study with Hybrid Knife, the heterogeneity decreased to 50% and the new pooled outcome revealed a significant improvement in 12‐month RFS associated with ERBT. Different energy could bring different outcomes in ERBT.^48^ Based on these findings, we concluded that ERBT was significantly associated with an improved 12‐month RFS. Based on the combined analysis of perioperative and survival outcomes, we suggest that ERBT is a safe surgical approach that may offer postoperative and RFS benefits for patients with NMIBC.

There were some limitations should be noticed. First, the 24‐month RFS did not pooled due to lack the data of RCTs. However, the 24‐month RFS outcomes of ERBT and cTURBT were found to be comparable, indicating that ERBT did not yield worse results in terms of 24‐month RFS. Second, we did not compare the RFS between laser and bipolar due to lack the data. This limitation maybe discussed in future when data is enough.

## 5 CONCLUSION

Using a combination of umbrella review and meta‐analysis, we demonstrated that ERBT had better or comparable perioperative outcome than cTURBT. Furthermore, NMIBC patients undergoing ERBT exhibited improved 3 and 12‐month RFS compared to those receiving cTURBT. We suggest that ERBT maybe a better surgical method for patients with NMIBC compared with cTURBT.

## AUTHOR CONTRIBUTIONS

**Deng‐xiong Li:** Conceptualization (lead); data curation (lead); formal analysis (lead); investigation (equal); methodology (lead); software (equal); visualization (equal); writing – original draft (equal). **Qing‐xin Yu:** Project administration (lead); resources (equal); supervision (equal); validation (equal). **Rui‐cheng Wu:** Formal analysis (equal); investigation (lead); methodology (equal); software (lead). **Jie Wang:** Validation (lead). **De‐chao Feng:** Conceptualization (equal); data curation (equal); formal analysis (lead); methodology (lead); visualization (equal); writing – original draft (equal). **Shi Deng:** Project administration (lead); resources (equal); supervision (equal); validation (equal); visualization (equal); writing – review and editing (lead).

## FUNDING INFORMATION

This research was funded by Chinese Scholarship Council (grant no. 202206240086). The funder had no role in the study design, data collection or analysis, preparation of the manuscript, or the decision to publish.

## CONFLICT OF INTEREST STATEMENT

None.

## ETHICS STATEMENT

This study is an umbrella review and meta‐analysis. Therefore, it does not require ethical review and approval.

## Supporting information


Table S1.

Table S2.

Table S3.

## Data Availability

All data from this study were downloaded from an online database. Therefore, everyone can get the data online. Further inquiries can be directed to the corresponding author.
