# Supplementary material for: Efficiency of transurethral en‐bloc resection vs. conventional transurethral resection for non‐muscle‐invasive bladder cancer: An umbrella review
Source: Cancer Med. 2024 May 31;13(11):e7323. doi: 10.1002/cam4.7323 (PMC11141332; doi:10.1002/cam4.7323)
Supplement: Supplementary file 2 — Table S2. [file CAM4-13-e7323-s002.docx]

**Supplementary Table 2. Methodological quality assessment of included meta-analyses using ROBIS**

A) Assessing relevance (phase I)

| **Author** | **Year** | **Patient** | **Intervention** | **Comparator** | **Outcome** | **Summarized of QOL** | **Rationale** |
| --- | --- | --- | --- | --- | --- | --- | --- |
| Wang_CW | 2023 | Low | Low | Low | Low | Low | There was no concern in assessing relevance part |
| Yanagisawa_T | 2022 | Low | Low | Low | Low | Low | There was no concern in assessing relevance part |
| Motlagh_RS | 2022 | Low | Low | Low | Low | Low | There was no concern in assessing relevance part |
| Li_ZY | 2022 | Low | Low | Low | Low | Low | There was no concern in assessing relevance part |
| Di_Y | 2022 | Low | Low | Low | Low | Low | There was no concern in assessing relevance part |
| Zhang_D | 2020 | Low | Low | Low | Low | Low | There was no concern in assessing relevance part |
| Yang_H | 2020 | Low | Low | Low | Low | Low | There was no concern in assessing relevance part |
| Wu_YP | 2016 | Low | Low | Low | Low | Low | There was no concern in assessing relevance part |
| Teoh_YJ | 2020 | Low | Low | Low | Low | Low | There was no concern in assessing relevance part |

*QOL; quality.

†If the answers to all signalling questions for a domain were “yes” or “probably yes,” then level of concern could be judged as low. If any signalling question was answered “no” or “probably no,” there was potential for concern about high bias.

B) Judging risk of bias

| **Author** | **Year** | **Phase II** | | | | **Phase III** | **Rationale** |
| --- | --- | --- | --- | --- | --- | --- | --- |
|  |  | **Domain1**  (Study eligibility criteria) | **Domain2**  (Identification and selection of studies) | **Domain3**  (Data collection and study appraisal) | **Domain4**  (Synthesis and findings) | **Risk of bias in the review** |  |
| Wang_CW | 2023 | High | Low | Low | High | High | 1. Restriction of included studies (language),  2. No information of heterogeneity |
| Yanagisawa_T | 2022 | Low | Low | Low | Low | Low | 1. There was no concern |
| Motlagh_RS | 2022 | Low | Low | Low | Low | Low | 1. There was no concern |
| Li_ZY | 2022 | Low | High | Low | Low | High | 1. Restriction of included studies (language) |
| Di_Y | 2022 | Low | High | Low | Low | High | 1. Restriction of included studies (language) |
| Zhang_D | 2020 | Low | High | Low | Low | High | 1. Restriction of included studies (language) |
| Yang_H | 2020 | Low | High | High | Low | High | 1. No information of risk bias assessment,  2. Restriction of included studies (language) |
| Wu_YP | 2016 | Low | High | High | Low | High | 1. Restriction of included studies (language),  2. No information of risk bias assessment |
| Teoh_YJ | 2020 | Low | High | Low | Low | High | 1. Restriction of included studies (language) |

†If the answers to all signalling questions for a domain were “yes” or “probably yes,” then level of concern could be judged as low. If any signalling question was answered “no” or “probably no,” there was potential for concern about high bias.
