# Supplementary material for: Efficiency of transurethral en‐bloc resection vs. conventional transurethral resection for non‐muscle‐invasive bladder cancer: An umbrella review
Source: Cancer Med. 2024 May 31;13(11):e7323. doi: 10.1002/cam4.7323 (PMC11141332; doi:10.1002/cam4.7323)
Supplement: Supplementary file 3 — Table S3. [file CAM4-13-e7323-s003.docx]

**Supplementary 3. GRADE results.**

**Question:** EBTR compared to cTURBT for detrusor muscle.

| **Certainty assessment** | | | | | | | **№ of patients** | | **Effect** | | **Certainty** | **Importance** |
| --- | --- | --- | --- | --- | --- | --- | --- | --- | --- | --- | --- | --- |
| **№ of studies** | **Study design** | **Risk of bias** | **Inconsistency** | **Indirectness** | **Imprecision** | **Other considerations** | **EBTR** | **cTURBT** | **Relative (95% CI)** | **Absolute (95% CI)** |  |  |
| **Wang_CW (2023)** | | | | | | | | | | | | |
| 13 | observational studies | not serious | not serious | not serious | serious^a^ | none | 766/926 (82.7%) | 719/1006 (71.5%) | **OR 3.85** (1.59 to 10.00) | **191 more per 1,000** (from 85 more to 247 more) | ⨁◯◯◯ Very low |  |
| **Yanagisawa_T (2022) RCT** | | | | | | | | | | | | |
| 4 | randomised trials | not serious | not serious | not serious | not serious | none | 241/271 (88.9%) | 185/272 (68.0%) | **RR 1.31** (1.19 to 1.43) | **211 more per 1,000** (from 129 more to 292 more) | ⨁⨁⨁⨁ High |  |
| **Yanagisawa_T (2022) NRCT** | | | | | | | | | | | | |
| 14 | observational studies | not serious | not serious | not serious | serious^a^ | none | 1065/1124 (94.8%) | 801/1115 (71.8%) | **RR 1.35** (1.21 to 1.50) | **251 more per 1,000** (from 151 more to 359 more) | ⨁◯◯◯ Very low |  |
| **Motlagh_RS (2022) RCT** | | | | | | | | | | | | |
| 5 | randomised trials | not serious | not serious | not serious | serious^a^ | none | 373/407 (91.6%) | 330/415 (79.5%) | **RR 1.17** (0.99 to 1.38) | **135 more per 1,000** (from 8 fewer to 302 more) | ⨁⨁⨁◯ Moderate |  |
| **Motlagh_RS (2022) NRCT** | | | | | | | | | | | | |
| 6 | observational studies | not serious | not serious | not serious | serious^a^ | none | 316/392 (80.6%) | 270/382 (70.7%) | **RR 1.16** (0.90 to 1.49) | **113 more per 1,000** (from 71 fewer to 346 more) | ⨁◯◯◯ Very low |  |
| **Li_ZY (2022)** | | | | | | | | | | | | |
| 4 | randomised trials | not serious | not serious | not serious | serious^a^ | none | 325/371 (87.6%) | 285/369 (77.2%) | **RR 2.38** (0.71 to 8.06) | **1,000 more per 1,000** (from 224 fewer to 1,000 more) | ⨁⨁⨁◯ Moderate |  |

**CI:** confidence interval; **OR:** odds ratio; **RR:** risk ratio

#### Explanations

a. The heterogeneity (I2) higher than 50%.

**Question:** EBTR compared to cTURBT for residual tumor rate.

| **Certainty assessment** | | | | | | | **№ of patients** | | **Effect** | | **Certainty** | **Importance** |
| --- | --- | --- | --- | --- | --- | --- | --- | --- | --- | --- | --- | --- |
| **№ of studies** | **Study design** | **Risk of bias** | **Inconsistency** | **Indirectness** | **Imprecision** | **Other considerations** | **EBTR** | **cTURBT** | **Relative (95% CI)** | **Absolute (95% CI)** |  |  |
| **Wang_CW (2023)** | | | | | | | | | | | | |
| 4 | observational studies | not serious | not serious | not serious | not serious | none | 16/149 (10.7%) | 54/189 (28.6%) | **OR 0.30** (0.16 to 0.57) | **179 fewer per 1,000** (from 226 fewer to 100 fewer) | ⨁⨁◯◯ Low |  |
| **Yanagisawa_T (2022)** | | | | | | | | | | | | |
| 4 | observational studies | not serious | not serious | not serious | not serious | none | 21/180 (11.7%) | 63/221 (28.5%) | **RR 0.47** (0.31 to 0.71) | **151 fewer per 1,000** (from 197 fewer to 83 fewer) | ⨁⨁◯◯ Low |  |
| **Li_ZY (2022)** | | | | | | | | | | | | |
| 2 | randomised trials | not serious | not serious | not serious | not serious | none | 4/199 (2.0%) | 14/193 (7.3%) | **OR 0.44** (0.08 to 0.77) | **39 fewer per 1,000** (from 66 fewer to 16 fewer) | ⨁⨁⨁⨁ High |  |

**CI:** confidence interval; **OR:** odds ratio; **RR:** risk ratio

**Question:** EBRT compared to cTURBT for operation time.

| **Certainty assessment** | | | | | | | **№ of patients** | | **Effect** | | **Certainty** | **Importance** |
| --- | --- | --- | --- | --- | --- | --- | --- | --- | --- | --- | --- | --- |
| **№ of studies** | **Study design** | **Risk of bias** | **Inconsistency** | **Indirectness** | **Imprecision** | **Other considerations** | **EBRT** | **cTURBT** | **Relative (95% CI)** | **Absolute (95% CI)** |  |  |
| **Wang_CW (2023)** | | | | | | | | | | | | |
| 21 | observational studies | not serious | not serious | not serious | serious^a^ | none | 1501 | 1679 | - | MD **0.08 lower** (1.87 lower to 1.72 higher) | ⨁◯◯◯ Very low |  |
| **Yanagisawa_T (2022)** | | | | | | | | | | | | |
| 9 | observational studies | not serious | not serious | not serious | serious^a^ | none | 643 | 631 | - | MD **5.38 higher** (0.33 higher to 10.42 higher) | ⨁◯◯◯ Very low |  |
| **Li_ZY (2022)** | | | | | | | | | | | | |
| 5 | randomised trials | not serious | not serious | not serious | serious^a^ | none | 470 | 457 | - | MD **1.78 higher** (0.94 lower to 4.5 higher) | ⨁⨁⨁◯ Moderate |  |
| **Yang_H (2020)** | | | | | | | | | | | | |
| 9 | observational studies | not serious | not serious | not serious | serious | none | 498 | 522 | - | SMD **0.05 SD higher** (0.23 lower to 0.32 higher) | ⨁◯◯◯ Very low |  |
| **Wu_YP (2016)** | | | | | | | | | | | | |
| 6 | observational studies | not serious | not serious | not serious | serious^a^ | none | 374 | 397 | - | MD **1.92 higher** (2.33 lower to 6.18 higher) | ⨁◯◯◯ Very low |  |
| **Teoh_YJ (2020)** | | | | | | | | | | | | |
| 10 | randomised trials | not serious | not serious | not serious | serious^a^ | none | 586 | 569 | - | MD **9.07 higher** (3.36 higher to 14.79 higher) | ⨁⨁⨁◯ Moderate |  |

**CI:** confidence interval; **MD:** mean difference; **SMD:** standardised mean difference

#### Explanations

a. The heterogeneity (I2) higher than 50%.

**Question:** EBRT compared to cTURBT for hospitalization time

| **Certainty assessment** | | | | | | | **№ of patients** | | **Effect** | | **Certainty** | **Importance** |
| --- | --- | --- | --- | --- | --- | --- | --- | --- | --- | --- | --- | --- |
| **№ of studies** | **Study design** | **Risk of bias** | **Inconsistency** | **Indirectness** | **Imprecision** | **Other considerations** | **EBRT** | **cTURBT** | **Relative (95% CI)** | **Absolute (95% CI)** |  |  |
| **Wang_CW (2023)** | | | | | | | | | | | | |
| 19 | observational studies | not serious | not serious | not serious | serious^a^ | none | 1338 | 1486 | - | MD **0.96 lower** (1.28 lower to 0.56 lower) | ⨁◯◯◯ Very low |  |
| **Li_ZY (2022)** | | | | | | | | | | | | |
| 6 | randomised trials | not serious | not serious | not serious | serious^a^ | none | 427 | 423 | - | MD **0.5 lower** (1.21 lower to 0.2 higher) | ⨁⨁⨁◯ Moderate |  |
| **Di_Y (2022)** | | | | | | | | | | | | |
| 15 | observational studies | not serious | not serious | not serious | serious^a^ | none | 918 | 912 | - | MD **0.95 lower** (1.55 lower to 0.34 lower) | ⨁◯◯◯ Very low |  |
| **Yang_H (2020)** | | | | | | | | | | | | |
| 9 | observational studies | not serious | not serious | not serious | serious^a^ | none | 498 | 522 | - | MD **0.88 lower** (1.57 lower to 0.19 lower) | ⨁◯◯◯ Very low |  |
| **Wu_YP (2016)** | | | | | | | | | | | | |
| 7 | observational studies | not serious | not serious | not serious | serious^a^ | none | 438 | 448 | - | MD **1.22 lower** (1.63 lower to 0.8 lower) | ⨁◯◯◯ Very low |  |

**CI:** confidence interval; **MD:** mean difference

#### Explanations

a. The heterogeneity (I2) higher than 50%.

**Question:** EBRT compared to cTURBT for catheterization time

| **Certainty assessment** | | | | | | | **№ of patients** | | **Effect** | | **Certainty** | **Importance** |
| --- | --- | --- | --- | --- | --- | --- | --- | --- | --- | --- | --- | --- |
| **№ of studies** | **Study design** | **Risk of bias** | **Inconsistency** | **Indirectness** | **Imprecision** | **Other considerations** | **EBRT** | **cTURBT** | **Relative (95% CI)** | **Absolute (95% CI)** |  |  |
| **Wang_CW (2023)** | | | | | | | | | | | | |
| 21 | observational studies | not serious | not serious | not serious | serious^a^ | none | 1440 | 1602 | - | MD **0.77 lower** (1.07 lower to 0.47 lower) | ⨁◯◯◯ Very low |  |
| **Yanagisawa_T (2022)** | | | | | | | | | | | | |
| 7 | observational studies | not serious | not serious | serious^b^ | serious^a^ | none | 448 | 441 | - | MD **1.07 lower** (1.63 lower to 0.51 lower) | ⨁◯◯◯ Very low |  |
| **Li_ZY (2022)** | | | | | | | | | | | | |
| 6 | randomised trials | not serious | not serious | not serious | serious^a^ | none | 427 | 423 | - | MD **0.41 lower** (1.09 lower to 0.28 higher) | ⨁⨁⨁◯ Moderate |  |
| **Di_Y (2022)** | | | | | | | | | | | | |
| 17 | observational studies | not serious | not serious | not serious | serious^a^ | none | 1093 | 1105 | - | MD **0.66 lower** (1.02 lower to 0.29 lower) | ⨁◯◯◯ Very low |  |
| **Yang_H (2020)** | | | | | | | | | | | | |
| 9 | observational studies | not serious | not serious | not serious | serious^a^ | none | 498 | 522 | - | MD **0.74 lower** (1.35 lower to 0.12 lower) | ⨁◯◯◯ Very low |  |
| **Wu_YP (2016** | | | | | | | | | | | | |
| 7 | observational studies | not serious | not serious | not serious | serious^a^ | none | 438 | 448 | - | MD **1.22 lower** (1.63 lower to 0.8 lower) | ⨁◯◯◯ Very low |  |

**CI:** confidence interval; **MD:** mean difference

#### Explanations

a. The heterogeneity (I2) higher than 50%.

b. Poor consistency of graphics.

**Question:** EBRT compared to cTURBT for bladder perforation?

| **Certainty assessment** | | | | | | | **№ of patients** | | **Effect** | | **Certainty** | **Importance** |
| --- | --- | --- | --- | --- | --- | --- | --- | --- | --- | --- | --- | --- |
| **№ of studies** | **Study design** | **Risk of bias** | **Inconsistency** | **Indirectness** | **Imprecision** | **Other considerations** | **EBRT** | **cTURBT** | **Relative (95% CI)** | **Absolute (95% CI)** |  |  |
| **Wang_CW (2023)** | | | | | | | | | | | | |
| 22 | observational studies | not serious | not serious | not serious | not serious | strong association | 8/1531 (0.5%) | 63/1569 (4.0%) | **OR 0.24** (0.11 to 0.44) | **30 fewer per 1,000** (from 36 fewer to 22 fewer) | ⨁⨁⨁◯ Moderate |  |
| **Yanagisawa_T (2022)** | | | | | | | | | | | | |
| 8 | observational studies | not serious | not serious | not serious | not serious | none | 1/654 (0.2%) | 31/643 (4.8%) | **OR 0.13** (0.05 to 0.34) | **42 fewer per 1,000** (from 46 fewer to 31 fewer) | ⨁⨁◯◯ Low |  |
| **Li_ZY (2022)** | | | | | | | | | | | | |
| 5 | randomised trials | not serious | not serious | not serious | not serious | none | 1/377 (0.3%) | 12/373 (3.2%) | **OR 0.17** (0.05 to 0.17) | **27 fewer per 1,000** (from 31 fewer to 27 fewer) | ⨁⨁⨁⨁ High |  |
| **Yang_H (2020)** | | | | | | | | | | | | |
| 5 | observational studies | not serious | not serious | not serious | not serious | none | 1/338 (0.3%) | 17/337 (5.0%) | **OR 0.15** (0.05 to 0.52) | **43 fewer per 1,000** (from 48 fewer to 24 fewer) | ⨁⨁◯◯ Low |  |
| **Wu_YP (2016)** | | | | | | | | | | | | |
| 4 | observational studies | not serious | not serious | not serious | not serious | none | 1/312 (0.3%) | 14/293 (4.8%) | **OR 0.14** (0.04 to 0.54) | **41 fewer per 1,000** (from 46 fewer to 21 fewer) | ⨁⨁◯◯ Low |  |
| **Teoh_YJ (2020)** | | | | | | | | | | | | |
| 5 | randomised trials | not serious | not serious | not serious | not serious | none | 4/370 (1.1%) | 18/353 (5.1%) | **OR 0.30** (0.11 to 0.83) | **35 fewer per 1,000** (from 45 fewer to 8 fewer) | ⨁⨁⨁⨁ High |  |

**CI:** confidence interval; **OR:** odds ratio

**Question:** EBRT compared to cTURBT for obturator nerve reflexe

| **Certainty assessment** | | | | | | | **№ of patients** | | **Effect** | | **Certainty** | **Importance** |
| --- | --- | --- | --- | --- | --- | --- | --- | --- | --- | --- | --- | --- |
| **№ of studies** | **Study design** | **Risk of bias** | **Inconsistency** | **Indirectness** | **Imprecision** | **Other considerations** | **EBRT** | **cTURBT** | **Relative (95% CI)** | **Absolute (95% CI)** |  |  |
| **Wang_CW (2023)** | | | | | | | | | | | | |
| 21 | observational studies | not serious | not serious | not serious | serious^a^ | strong association | 46/1406 (3.3%) | 195/1421 (13.7%) | **OR 0.13** (0.06 to 0.19) | **117 fewer per 1,000** (from 128 fewer to 108 fewer) | ⨁⨁◯◯ Low |  |
| **Li_ZY (2022)** | | | | | | | | | | | | |
| 4 | randomised trials | not serious | not serious | not serious | not serious | none | 0/307 (0.0%) | 50/308 (16.2%) | **OR 0.03** (0.01 to 0.13) | **157 fewer per 1,000** (from 160 fewer to 138 fewer) | ⨁⨁⨁⨁ High |  |
| **Yang_H (2020)** | | | | | | | | | | | | |
| 7 | observational studies | not serious | not serious | not serious | not serious | none | 0/443 (0.0%) | 69/438 (15.8%) | **OR 0.04** (0.01 to 0.12) | **150 fewer per 1,000** (from 156 fewer to 136 fewer) | ⨁⨁◯◯ Low |  |
| **Wu_YP (2016)** | | | | | | | | | | | | |
| 4 | observational studies | not serious | not serious | not serious | not serious | none | 0/319 (0.0%) | 42/308 (13.6%) | **OR 0.04** (0.01 to 0.15) | **130 fewer per 1,000** (from 135 fewer to 113 fewer) | ⨁⨁◯◯ Low |  |

**CI:** confidence interval; **OR:** odds ratio

#### Explanations

a. The heterogeneity (I2) higher than 50%.

**Question:** EBRT compared to cTURBT for bladder irritation

| **Certainty assessment** | | | | | | | **№ of patients** | | **Effect** | | **Certainty** | **Importance** |
| --- | --- | --- | --- | --- | --- | --- | --- | --- | --- | --- | --- | --- |
| **№ of studies** | **Study design** | **Risk of bias** | **Inconsistency** | **Indirectness** | **Imprecision** | **Other considerations** | **EBRT** | **cTURBT** | **Relative (95% CI)** | **Absolute (95% CI)** |  |  |
| **Wang_CW (2023)** | | | | | | | | | | | | |
| 5 | observational studies | not serious | not serious | not serious | serious^a^ | none | 58/289 (20.1%) | 120/276 (43.5%) | **OR 0.22** (0.08 to 0.60) | **290 fewer per 1,000** (from 377 fewer to 119 fewer) | ⨁◯◯◯ Very low |  |
| **Di_Y (2022)** | | | | | | | | | | | | |
| 8 | observational studies | not serious | not serious | not serious | serious^a^ | none | 501 | 508 | - | MD **6.06 lower** (9.45 lower to 2.67 lower) | ⨁◯◯◯ Very low |  |
| **Yang_H (2020)** | | | | | | | | | | | | |
| 3 | observational studies | not serious | not serious | not serious | serious^a^ | none | 124 | 130 | - | MD **1.6 lower** (3.47 lower to 0.28 lower) | ⨁◯◯◯ Very low |  |
| **Wu_YP (2016)** | | | | | | | | | | | | |
| 4 | observational studies | not serious | not serious | not serious | serious^a^ | none | 1/312 (0.3%) | 14/293 (4.8%) | **OR 0.14** (0.04 to 0.54) | **41 fewer per 1,000** (from 46 fewer to 21 fewer) | ⨁◯◯◯ Very low |  |
| **Teoh_YJ (2020)** | | | | | | | | | | | | |
| 2 | randomised trials | not serious | not serious | not serious | serious^a^ | none | 102 | 93 | - | MD **7.24 lower** (9.29 lower to 5.2 lower) | ⨁⨁⨁◯ Moderate |  |

**CI:** confidence interval; **MD:** mean difference; **OR:** odds ratio

#### Explanations

a. The heterogeneity (I2) higher than 50%.

**Question:** EBRT compared to cTURBT for 3 months RFS

| **Certainty assessment** | | | | | | | **№ of patients** | | **Effect** | | **Certainty** | **Importance** |
| --- | --- | --- | --- | --- | --- | --- | --- | --- | --- | --- | --- | --- |
| **№ of studies** | **Study design** | **Risk of bias** | **Inconsistency** | **Indirectness** | **Imprecision** | **Other considerations** | **EBRT** | **cTURBT** | **Relative (95% CI)** | **Absolute (95% CI)** |  |  |
| **Wang_CW (2023)** | | | | | | | | | | | | |
| 4 | observational studies | not serious | not serious | not serious | not serious | none | 25/312 (8.0%) | 73/462 (15.8%) | **OR 0.50** (0.30 to 0.81) | **72 fewer per 1,000** (from 105 fewer to 26 fewer) | ⨁⨁◯◯ Low |  |
| **Motlagh_RS (2022) RCT** | | | | | | | | | | | | |
| 4 | randomised trials | not serious | not serious | not serious | not serious | none | 8/297 (2.7%) | 16/319 (5.0%) | **RR 0.57** (0.25 to 1.27) | **22 fewer per 1,000** (from 38 fewer to 14 more) | ⨁⨁⨁⨁ High |  |
| **Motlagh_RS (2022) NRCT** | | | | | | | | | | | | |
| 5 | observational studies | not serious | not serious | not serious | not serious | none | 22/358 (6.1%) | 47/352 (13.4%) | **RR 0.46** (0.29 to 0.73) | **72 fewer per 1,000** (from 95 fewer to 36 fewer) | ⨁⨁◯◯ Low |  |
| **Li_ZY (2022)** | | | | | | | | | | | | |
| 6 | randomised trials | not serious | not serious | not serious | not serious | none | 8/512 (1.6%) | 16/510 (3.1%) | **OR 0.50** (0.21 to 1.17) | **15 fewer per 1,000** (from 25 fewer to 5 more) | ⨁⨁⨁⨁ High |  |

**CI:** confidence interval; **OR:** odds ratio; **RR:** risk ratio

**Question:** EBRT compared to cTURBT for 1 year RFS

| **Certainty assessment** | | | | | | | **№ of patients** | | **Effect** | | **Certainty** | **Importance** |
| --- | --- | --- | --- | --- | --- | --- | --- | --- | --- | --- | --- | --- |
| **№ of studies** | **Study design** | **Risk of bias** | **Inconsistency** | **Indirectness** | **Imprecision** | **Other considerations** | **EBRT** | **cTURBT** | **Relative (95% CI)** | **Absolute (95% CI)** |  |  |
| **Wang_CW (2023)** | | | | | | | | | | | | |
| 9 | observational studies | not serious | not serious | not serious | not serious | none | 87/452 (19.2%) | 101/489 (20.7%) | **OR 0.79** (0.49 to 1.27) | **36 fewer per 1,000** (from 93 fewer to 42 more) | ⨁⨁◯◯ Low |  |
| **Yanagisawa_T (2022)** | | | | | | | | | | | | |
| 6 | observational studies | not serious | not serious | not serious | not serious | none | 88/380 (23.2%) | 87/364 (23.9%) | **RR 0.98** (0.76 to 1.26) | **5 fewer per 1,000** (from 57 fewer to 62 more) | ⨁⨁◯◯ Low |  |
| **Motlagh_RS (2022) RCT** | | | | | | | | | | | | |
| 7 | randomised trials | not serious | not serious | not serious | not serious | none | 85/504 (16.9%) | 95/513 (18.5%) | **OR 0.89** (0.69 to 1.15) | **17 fewer per 1,000** (from 50 fewer to 22 more) | ⨁⨁⨁⨁ High |  |
| **Motlagh_RS (2022) NRCT** | | | | | | | | | | | | |
| 4 | observational studies | not serious | not serious | not serious | not serious | none | 18/178 (10.1%) | 31/191 (16.2%) | **OR 0.56** (0.33 to 0.96) | **64 fewer per 1,000** (from 102 fewer to 5 fewer) | ⨁⨁◯◯ Low |  |
| **Li_ZY (2022)** | | | | | | | | | | | | |
| 7 | randomised trials | not serious | not serious | not serious | not serious | none | 84/576 (14.6%) | 88/566 (15.5%) | **OR 0.91** (0.56 to 1.29) | **12 fewer per 1,000** (from 62 fewer to 36 more) | ⨁⨁⨁⨁ High |  |
| **Di_Y (2022)** | | | | | | | | | | | | |
| 14 | observational studies | not serious | not serious | not serious | not serious | none | 123/825 (14.9%) | 160/934 (17.1%) | **OR 0.79** (0.61 to 1.04) | **31 fewer per 1,000** (from 59 fewer to 6 more) | ⨁⨁◯◯ Low |  |
| **Zhang_D (2020)** | | | | | | | | | | | | |
| 9 | observational studies | not serious | not serious | not serious | not serious | none | 81/479 (16.9%) | 103/512 (20.1%) | **OR 0.77** (0.55 to 1.07) | **39 fewer per 1,000** (from 80 fewer to 11 more) | ⨁⨁◯◯ Low |  |
| **Teoh_YJ (2020)** | | | | | | | | | | | | |
| 5 | randomised trials | not serious | not serious | not serious | not serious | none | 63/322 (19.6%) | 72/300 (24.0%) | **RR 0.82** (0.56 to 1.19) | **43 fewer per 1,000** (from 106 fewer to 46 more) | ⨁⨁⨁⨁ High |  |

**CI:** confidence interval; **OR:** odds ratio; **RR:** risk ratio

**Question:** EBRT compared to cTURBT for 2 years RFS

| **Certainty assessment** | | | | | | | **№ of patients** | | **Effect** | | **Certainty** | **Importance** |
| --- | --- | --- | --- | --- | --- | --- | --- | --- | --- | --- | --- | --- |
| **№ of studies** | **Study design** | **Risk of bias** | **Inconsistency** | **Indirectness** | **Imprecision** | **Other considerations** | **EBRT** | **cTURBT** | **Relative (95% CI)** | **Absolute (95% CI)** |  |  |
| **Wang_CW (2023)** | | | | | | | | | | | | |
| 9 | observational studies | not serious | not serious | not serious | not serious | none | 87/452 (19.2%) | 101/489 (20.7%) | **OR 0.79** (0.49 to 1.27) | **36 fewer per 1,000** (from 93 fewer to 42 more) | ⨁⨁◯◯ Low |  |
| **Yanagisawa_T (2022)** | | | | | | | | | | | | |
| 4 | observational studies | not serious | not serious | not serious | not serious | none | 38/295 (12.9%) | 44/287 (15.3%) | **RR 0.85** (0.55 to 1.23) | **23 fewer per 1,000** (from 69 fewer to 35 more) | ⨁⨁◯◯ Low |  |
| **Li_ZY (2022)** | | | | | | | | | | | | |
| 5 | randomised trials | not serious | not serious | not serious | not serious | none | 97/449 (21.6%) | 100/436 (22.9%) | **OR 0.89** (0.63 to 1.26) | **20 fewer per 1,000** (from 71 fewer to 43 more) | ⨁⨁⨁⨁ High |  |
| **Di_Y (2022)** | | | | | | | | | | | | |
| 18 | observational studies | not serious | not serious | not serious | not serious | strong association | 174/994 (17.5%) | 282/1084 (26.0%) | **OR 0.63** (0.50 to 0.78) | **79 fewer per 1,000** (from 111 fewer to 45 fewer) | ⨁⨁⨁◯ Moderate |  |
| **Zhang_D (2020)** | | | | | | | | | | | | |
| 14 | observational studies | not serious | not serious | not serious | not serious | none | 134/762 (17.6%) | 200/797 (25.1%) | **OR 0.62** (0.48 to 0.80) | **79 fewer per 1,000** (from 112 fewer to 40 fewer) | ⨁⨁◯◯ Low |  |
| **Yang_H (2020)** | | | | | | | | | | | | |
| 7 | observational studies | not serious | not serious | not serious | not serious | none | 90/393 (22.9%) | 0.0% | **-0.08** (-0.14 to -0.02) | **-- per 1,000** (from -- to --) | ⨁⨁◯◯ Low |  |
| **Wu_YP (2016)** | | | | | | | | | | | | |
| 6 | observational studies | not serious | not serious | not serious | not serious | none | 86/367 (23.4%) | 119/377 (31.6%) | **OR 0.66** (0.47 to 0.92) | **82 fewer per 1,000** (from 137 fewer to 18 fewer) | ⨁⨁◯◯ Low |  |
| **Teoh_YJ (2020)** | | | | | | | | | | | | |
| 2 | randomised trials | not serious | not serious | not serious | not serious | none | 17/135 (12.6%) | 20/127 (15.7%) | **RR 0.79** (0.44 to 1.42) | **33 fewer per 1,000** (from 88 fewer to 66 more) | ⨁⨁⨁⨁ High |  |

**CI:** confidence interval; **OR:** odds ratio; **RR:** risk ratio
